# Differences in Patterns of Alcohol Use and Sexual Risk-Taking Behaviours Among Sexually Active Australian and Overseas-Born Domestic and International University Students in Australia

**DOI:** 10.3390/ijerph23050547

**Published:** 2026-04-23

**Authors:** Alex Leong, Erich C. Fein, Kirstie Daken, Judith A. Dean, Sara F. E. Bell, Joseph Debattista, Armin Ariana, Kathryn Elizabeth Wenham, Joanne Durham, Charles F. Gilks, Zhihong Gu, Amy B. Mullens

**Affiliations:** 1 School of Health, Psychological and Medical Sciences, Institute for Health, University of Southern Queensland, Toowoomba, QLD 4350, Australia; erich.fein@unisq.edu.au (E.C.F.); kirstie.daken@unisq.edu.au (K.D.); 2 School of Health, University of the Sunshine Coast, Sippy Downs, QLD 4558, Australia; 3 School of Public Health, Faculty of Health, Medicine and Behavioural Sciences, The University of Queensland, Brisbane, QLD 4000, Australia; j.dean4@uq.edu.au (J.A.D.); sara.bell@uq.edu.au (S.F.E.B.); 4 Metro North Public Health Unit, Metro North Hospital and Health Service, Windsor, Brisbane, QLD 4030, Australia; 5 School of Medicine and Dentistry, Griffith University, Gold Coast, QLD 4215, Australia; a.ariana@griffith.edu.au; 6 School of Public Health and Social Work, Queensland University of Technology, Kelvin Grove, Brisbane, QLD 4059, Australia; 7 Ethnic Communities Council, West End, QLD 4101, Australia

**Keywords:** alcohol use, sexual risk-taking, behaviours, university students

## Abstract

**Highlights:**

**Public health relevance—How does this work relate to a public health issue?**

- Alcohol use has been linked to sexual risk-taking among young people in Australia.
- Young people substantially represent sexually transmissible infection (STI) cases.

**Public health significance—Why is this work of significance to public health?**

- This is the first study to compare alcohol use and sexual risk-taking across Australian-born domestic, overseas-born domestic, and international students.
- Australian-born domestic students were more likely to consume alcohol at high-risk levels.

**Public health implications—What are the key implications or messages for practitioners, policy makers and/or researchers in public health?**

- Differences in sexual risk-taking were fully mediated by harmful alcohol use.
- The association between harmful drinking and sexual risk-taking was strongest among students aged 20–24.
- Culturally responsive and age-targeted interventions are important for informing practice and policy.

**Abstract:**

Alcohol use has been linked to sexual risk-taking behaviour, particularly among young people in Australia, who are also substantially represented in sexually transmissible infection (STI). While research on alcohol use and sexual risk-taking among university students in Australia exists, no studies outside recent Tertiary Students Sexual and Reproductive Health Survey (TSSHS) publications have distinguished between Australian-born and overseas-born domestic students, despite evidence that migrant populations may show different alcohol use and sexual behaviour patterns. Using data from the TSSHS and a cross-sectional anonymous online survey of university-enrolled students, this study is the first to compare sexually active Australian-born domestic, overseas-born domestic, and international students on alcohol use and sexual risk-taking. Findings align with past research, with Australian-born domestic students being more likely to consume alcohol at high-risk levels than international and overseas-born domestic students. Differences in sexual risk-taking behaviours between the three enrolment groups were fully mediated by harmful alcohol use, indicating an indirect effect between group membership and sexual risk-taking. Age moderated this mediation, with the association between harmful drinking and sexual risk-taking strongest among students aged 20–24, compared with younger and older groups.

## 1. Introduction

Young people attending university in Australia have been reported to engage in behaviours that place them at an increased risk of sexually transmissible infection (STIs) and other negative sexual and reproductive health (SRH) outcomes. For example, Whatnall et al. [1] report that out of 3077 sexually active students from an Australian university, 33.4% never use condoms and 19% do not use contraception. Another study involving university students aged 18 to 31 attending an Australian regional university reported those with more than six sexual partners in the past 12 months were the least likely to use condoms during sex, as compared to other student groups with less than 6 sexual partners in the past 12 months [2].

University students’ SRH-related risks vary by background and enrolment status. For example, international students studying at Australian universities have been reported to be at increased risk of adverse SRH outcomes compared with domestic students [3,4,5]. Douglass et al. [6] also found Chinese international students were more likely to use less effective methods of contraception, such as condoms and emergency contraception, as compared to domestic students. Chinese international students were however less likely to report risky sexual behaviours compared with domestic students [6]. While international students report lower sexual risk-taking behaviours, reports of poorer STI knowledge and SRH service use among international students could increase their risk of adverse SRH outcomes [5,7]. Barriers to accessing SRH services, such as embarrassment, judgment from health professionals, language, and perceived stigma associated with STIs or sexual orientation, further increase risks of adverse SRH outcomes for international students [8]. Acculturative stressors associated with adjusting to a new culture and environment, as well as differences in cultural norms in Western contexts which can be sexually liberating for international students both contribute to the likelihood of sexual exploration when abroad [9]. For example, international students from Asia and Sub-Saharan Africa studying in Australia are more likely to engage in non-committal or premarital sex, a cultural norm in Australia but not in their home countries [10,11,12]. Compared with domestic students, lower sexual health knowledge among international students increases their vulnerability to poorer SRH outcomes during this period of change in sexual behaviour and exploration [9].

Culture-related stressors and norms may also influence the SRH risk of domestic overseas-born students. Domestic overseas-born university students have also been found to report lower rates of testing compared with domestic Australian-born university students [7]. A systematic review of SRH literacy among young people from refugee and migrant backgrounds aged 16 to 24 years living in Australia found that SRH knowledge increases with time spent in Australia; however, sociocultural beliefs and contextual factors create barriers in accessing SRH information and services [13]. Sociocultural factors such as a lack of discussion of SRH topics with family members and among migrant and refugee communities limit access to SRH information. Young people from migrant and refugee backgrounds living in Australia rely on school-based education for SRH information [14]. These nuances and diversity in SRH knowledge, behaviours, and service use warrant further research to better understand the diverse needs of students based on their enrolment groups [7]. Despite growing research on sexual health and alcohol use among student groups in Australia, no studies have differentiated between overseas-born domestic students and international students specifically and examined how alcohol use may influence sexual risk-taking behaviours across these groups. Understanding these differences helps to clarify the potential influence of migration background, acculturative stressors, and sociocultural norms on sexual health and risk-taking behaviours, which may inform targeted health promotion and sexual health interventions within Australian university settings.

## 2. Alcohol-Related Harms, Age, and Acculturation Stressors

Research examining alcohol use and sexual risk-taking behaviours among young adults has identified several behavioural and contextual factors that may influence risk exposure. Sexual risk-taking behaviours are often conceptualised through behaviours such as condom use, number of sexual partners, and types of sexual activity, including vaginal, anal, and oral sex [15,16]. The frequency of these behaviours and the context in which they occur are important in determining overall risk exposure to STIs and HIV transmission [15]. However, there is no ‘gold standard’ measure of sexual risk-taking behaviours, as existing measures are often developed to address specific project needs and may vary depending on contextual factors such as partnership status, HIV status, and the use of protective strategies such as antiretroviral therapy or pre-exposure prophylaxis [17]. These findings highlight the importance of contextualising sexual risk-taking behaviours when examining STI risk among young adults.

Australian students are also at risk of alcohol-related disease or injury. For example, an online cross-sectional study found that 80% consumed alcohol in the past 12 months, with 50% of students drinking more than the recommended two standard drinks/day as per the Australian National Health and Medical Research Council (NHMRC) Alcohol Guidelines for lifetime risk of alcohol-related disease or injury [18]. Age is a significant influencing factor on alcohol consumption [18]. Younger university students (aged 18 to 30 years) are more likely to drink more than two standard drinks a day or experience alcohol-related blackouts compared with their older peers (aged 31 to 50 years). A systematic review of substance use and impact on sexual risk behaviour within United States and Canadian populations identified a relationship between alcohol consumption and sexual risk-taking behaviours among young adults. Studies included in the review found that alcohol intoxication can impair cognitive functioning and increase the likelihood of engaging in risky sexual behaviours, including unprotected sex and sex with multiple partners [19].

Newly arrived migrant and refugee people in Australia use alcohol as their main coping strategy to manage acculturation and resettlement stressors [20]. International students increase their alcohol consumption on arrival to Australia [21] but are less likely to drink amounts that are above the Australian NHMRC Alcohol Guidelines of lifetime risk, with reduced likelihood of experiencing alcohol-related harms compared to domestic students [18,22]. Nevertheless, international students in Australia experiencing mental health and other related challenges engage in health-compromising behaviours, including smoking, substance, and alcohol use to cope, highlighting the potential role of acculturative stressors on health outcomes [23]. Qualitative research findings are consistent with the impact of acculturative stressors on health outcomes and behaviours, identifying stress of transitioning to life in Australia, including adapting to social expectations, which may differ from their ethnic heritage, which increase alcohol consumption [24].

Sexual risk-taking behaviours associated with alcohol use among Australian sub-groups, including adolescents [25], young adults [26], and female university students [27], are reported. Alcohol use has also been linked to university students reporting regret about engaging in sexual activity [18]. However, differences between student group membership (Australian-born domestic university students, overseas-born domestic university students, and international students), sexual risk-taking behaviours, and the role of alcohol use in mediating the relationship between these factors are not understood.

Young people in general represent a substantial proportion of STI notifications in Australia with two-thirds of chlamydia notifications reported among 15- to 29-year-olds in 2022 [28]. This, combined with the diversity of sexual experiences, sexual health knowledge, and sexual health service use reported among Australian-born domestic students, overseas-born domestic students, and international students [7,9] and the lack of understanding of their relationship with the role of alcohol use supports the need for further examination of these factors.

**Figure 1:**
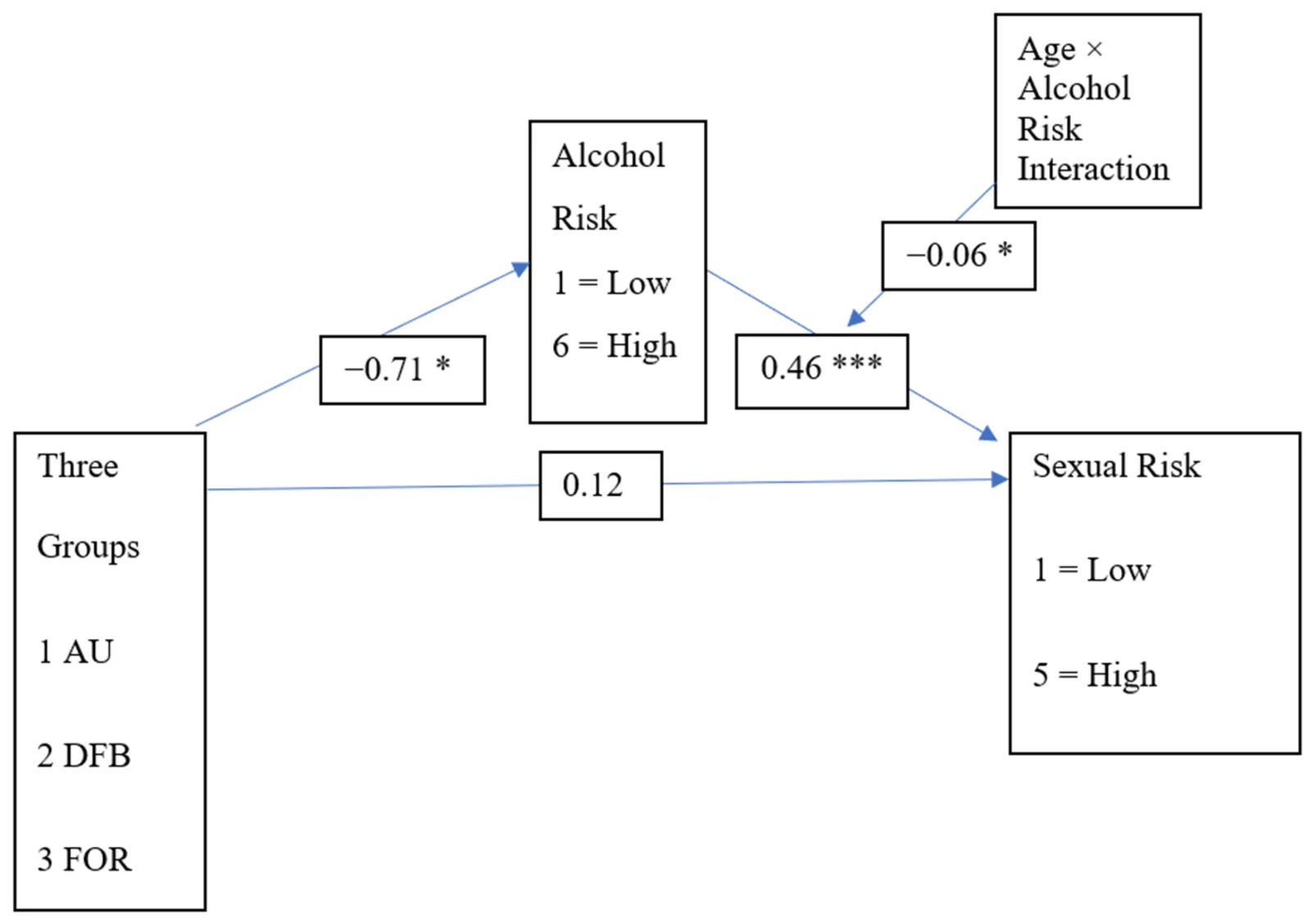
Process Model 14, groups by alcohol, sexual risk, and age. *** *p* < 0.01, * *p* < 0.05, *n* = 3325. Student groups were coded as 1 = Australian-born domestic students, 2 = domestic students who were born in countries other than Australia, 3 = international students.

Age has also been identified as an important influencing factor on alcohol consumption and associated harms [18], which is also hypothesised to account for differences in sexual risk-taking behaviour and alcohol use in the moderated mediation model. In accordance with the above relationships between alcohol use and sexual risk-taking behaviours, particularly risk-taking behaviours associated with alcohol use among Australian sub-groups, including young adults [26] and female university students [27], the potential role of acculturative stressors on health outcomes [23] and the particular role of alcohol use with migrants [20], we developed the model presented in Figure 1 of explanatory mechanisms to address differences between student groups in alcohol use and sexual risk-taking behaviours. With respect to mechanisms, we included alcohol use as the main mediator to explain the group differences and we also included the factor of age in the model as a moderating path, based on the literature showing it to be an important influencing factor on alcohol consumption and associated harms [18].

The present study aims to examine differences in alcohol use patterns and sexual risk-taking behaviours among Australian-born domestic students, overseas-born domestic students, and international students studying at Australian universities. Specifically, this study aims to: (1) identify differences in sexual risk-taking behaviours among the three student groups as mediated by alcohol use patterns and (2) examine if the effect between alcohol use patterns and sexual risk-taking behaviours among these groups is moderated by age. Therefore, it is hypothesised that:

**H1.**  *Membership in the three student groups will be associated with risky drinking behaviour, sexual risk-taking behaviour, and age.*

In a moderated mediation model (see Figure 1) depicting the relationships between sexual risk-taking behaviour and membership in the three student groups, with risky drinking as a mediator, and age as a second-stage moderator, the following findings are anticipated:

**H2A.**  *There will not be a direct relationship between student group membership and sexual risk-taking behaviour.*

**H2B.**  *There will be an indirect relationship between student group membership and sexual risk-taking behaviour which is mediated by risky drinking behaviour.*

**H2C.**  *The indirect relationship between student group membership and sexual risk-taking behaviour which is mediated by risky drinking behaviour will be moderated by age as a second-stage moderator.*

## 3. Methods

This study investigates the direct and indirect relationships between student group membership and sexual risk-taking behaviour, with alcohol use as a mediator, and age as a second-stage moderator. We accounted for the effect of age using a moderated mediation model (see Figure 1). Data from the Tertiary Student Sexual and Reproductive Health (TSSRH) survey, a cross-sectional anonymous online survey conducted between July and September 2019, was used to complete this analysis.

The TSSRH survey, a cross-sectional anonymous online survey conducted between July and September 2019, aims to understand the sexual and reproductive health knowledge, attitudes, behaviours of students and related factors influencing these behaviours such as substance use, mental health and student enrolment status in South-East Queensland. Primary ethics approval was granted by The University of Queensland (UQ) Human Research Ethics Committee (HREC 2018002579). It formed the basis for secondary, ratifying ethical approvals from the Human Research Ethics Committees at partnering universities (Queensland University of Technology (QUT), University of Southern Queensland (USQ), Griffith University (GU) and the University of the Sunshine Coast (USC).

### 3.1. Procedure

The TSSRH survey methods are reported elsewhere [3,4,5,7,8,29]. Briefly, convenience sampling using a range of active recruitment strategies was used to promote recruitment across five South-East Queensland universities. Recruitment strategies included social media posts, peer recruiters, printed material distributed at on-campus student events, and permission was gained to directly email the full student cohort enrolled at three of the partnering institutions. Targeted advertising to promote inclusion of priority populations (e.g., international, LGBTQIA+ students) was applied and peer recruiters from diverse socio-cultural and gender backgrounds—trained to ensure no coercive practices—were used to further assist with recruitment diversity. Snowball sampling through sharing of the survey among social or study networks was also promoted. Using the Australian Bureau of Statistics’ sample size calculator, a target sample size of 1400–1415 students was calculated [30].

Engagement was incentivised through an opt-in prize draw for one of five $100 grocery e-vouchers. Students were directed to the study website to access the participant information sheet. Students choosing to participate were required to answer screening questions to ensure eligibility (inclusion criteria: aged 18 years or older; current Queensland-based university enrolment; exclusion criteria: under 18 years and enrolled in University located in another state/jurisdiction/country) prior to providing informed consent via the online registration portal and accessing the full survey. Consent was further implied by the voluntary completion of the survey. The survey, developed by the partnering universities and SRH community organisations, and based on previously validated questions, was hosted on a University of Queensland server and delivered using REDCap (Research Electronic Data Capture), a secure web application used to build and manage surveys and store data. The survey, which included multiple-choice, Likert-type, and short-answer open-ended responses, was conducted in English (based on the assumption that English proficiency is required to study at Australian universities) and on average took 30–40 min to complete.

### 3.2. Participants

The focus of the present study is on sexual risk behaviours and alcohol use scores among Australian-born domestic students, overseas-born domestic students and international students. Student group membership was categorised by participant-reported enrolment status (domestic student or international student) and country of birth, creating three distinct categories (domestic Australian-born; domestic overseas-born; and international (overseas-born). For this study, only responses of students who were sexually active in the past 12 months and fully answered alcohol use questions were included for analysis (*n* = 3325, 77.5% of the 4291 respondents who completed the TSSRH survey).

Regarding distribution across student groups, of the total 3325 respondents, 2530 domestic Australian-born respondents constituted 76.1% of the sample, 349 domestic overseas-born respondents constituted 10.5% of the sample; and 446 international student respondents constituted 13.4% of the sample. Within our analyses, the student group variable was coded as 1 = Australian-born domestic students, 2 = domestic students who were born in countries other than Australia, 3 = international students. As this was a pilot study using convenience sampling, there was not a target sample size. The multiple recruitment strategies were designed to target these populations and consider priority groups known to be at an increased risk of STI.

Participant age was measured using six age categories. Of the total 3325 respondents, the frequencies were as follows: 18–19 years of age (25.8%), 20–24 years of age (45.5%), 25–29 years of age (15.2%), 30–34 years of age (6%), 35–39 years of age (3.5%), and 40 years of age or older (3.9%).

### 3.3. Measures

#### 3.3.1. Sexual Risk Index (SRI)

The current study selected four of the nine items included in the TSSHS, which constituted different types of risky sexual behaviours with regular sexual partners. These sexual risk-taking items were: (1) “How many different regular vaginal sex partners have you had in the last 12 months,” (2) “How often do you use condoms when you have vaginal sex,” (3) “How many different regular anal sex partners have you had in the last 12 months,” (4) “How often do you use condoms when you have anal sex.” The second and fourth items requiring graded (ordered) responses, e.g., ‘Not Usually’ or ‘Usually’, were assigned points ranging from zero to five with higher values reflecting greater frequency and amounts. Using all four items, we produced the Sexual Risk Index used in this study, which is a sum of these four items. This Sexual Risk Index had a mean of 5.69 (SD = 4.45).

#### 3.3.2. Alcohol Risk Index (ARI)

The alcohol use scale used a composite score, which was derived from responses on the alcohol use items in the TSSHS. These items determined if participants had used alcohol in their lifetime and alcohol use, amount and frequency in the past three months. In the present study, we calculated an Alcohol Risk Index composite measure based on a mean of the following four items: (1) “How often do you have sex when you have used alcohol,” (2) “In the past three months, how often did you usually have a drink containing alcohol,” (3) “How many standard drinks containing alcohol do you have on a typical day when you are drinking,” and (4) “In the past three months, how often have you had six or more standard drinks on one occasion.” The first, second and fourth items require scaling of ordered responses (e.g., ‘Weekly’ or ‘Monthly’; ‘Three to Four’ or ‘Five to Six’) and were assigned points ranging from zero to five with higher values reflecting greater frequency and amounts. This composite measure displayed a range from 0 to 17 with a mean of 5.91 (SD = 3.66).

### 3.4. Analysis

Data analyses were conducted using Statistical Package for Social Sciences (SPSS) version 29.0.1 software. Visual inspection of histograms and values of skewness and kurtosis were examined to inspect multivariate normality. All variables were approximately normal with values of skewness and kurtosis except for the Alcohol Risk Index, which displayed a kurtosis value of 3.0. However, this value was very close to traditional limits of +/− 2.0 [31]. Nevertheless, we used bootstrapping (BCa) methods to correct for biased and/or skewed distributions [31]. We used multivariate moderation and mediation analysis via the Hayes Process Macro to simultaneously compare direct and indirect effects for sexual risk-taking behaviour on student group, mediated by risky alcohol use, and moderated by age (dependent variables) across the three student groups (independent variable). See Figure 1 for the full model. Process Model 14 was employed using the bias-corrected and accelerated bootstrapping (BCa) method to correct for biased and/or skewed distributions [32].

## 4. Results

### Scores by Student Group and Correlations

Scoring of the Sexual Risk Index and Alcohol Risk Index and age category by student group is summarised in Table 1 below. A correlation matrix for study variables across all student groups is summarised in Table 2 below. The correlation matrix reveals significant, small to moderate correlation effect sizes between most variables (*r* = 0.04 to 0.27), except for the non-significant association between student group and sexual risk (*r* = −0.01).

## 5. Moderated Mediation Analyses

Moderation mediation analyses were conducted in line with Figure 1 based on bias-corrected bootstrapped confidence intervals with 5000 resamples. Within these analyses we posited a mediation effect for Alcohol Risk between group members and Sexual Risk based on risk-taking with regular partners as reported in Table 1 and Table 2. In addition, we expected to see a second-stage moderation effect for age on the relationship between the mediator, alcohol risk, and the outcome, sexual risk. The sample size for the moderation analyses was *n* = 3325. Because of some degree of conceptual overlap between alcohol risk and risky sexual activity, namely based inclusion within the Alcohol Risk Index of an item directly linking alcohol use to sexual activity, we examined the VIF statistic for this path in the model. The VIF value for the path between alcohol risk and sexual risk, as moderated by age, was VIF = 1.86, indicating that multicollinearity was not a problem.

**Figure 2:**
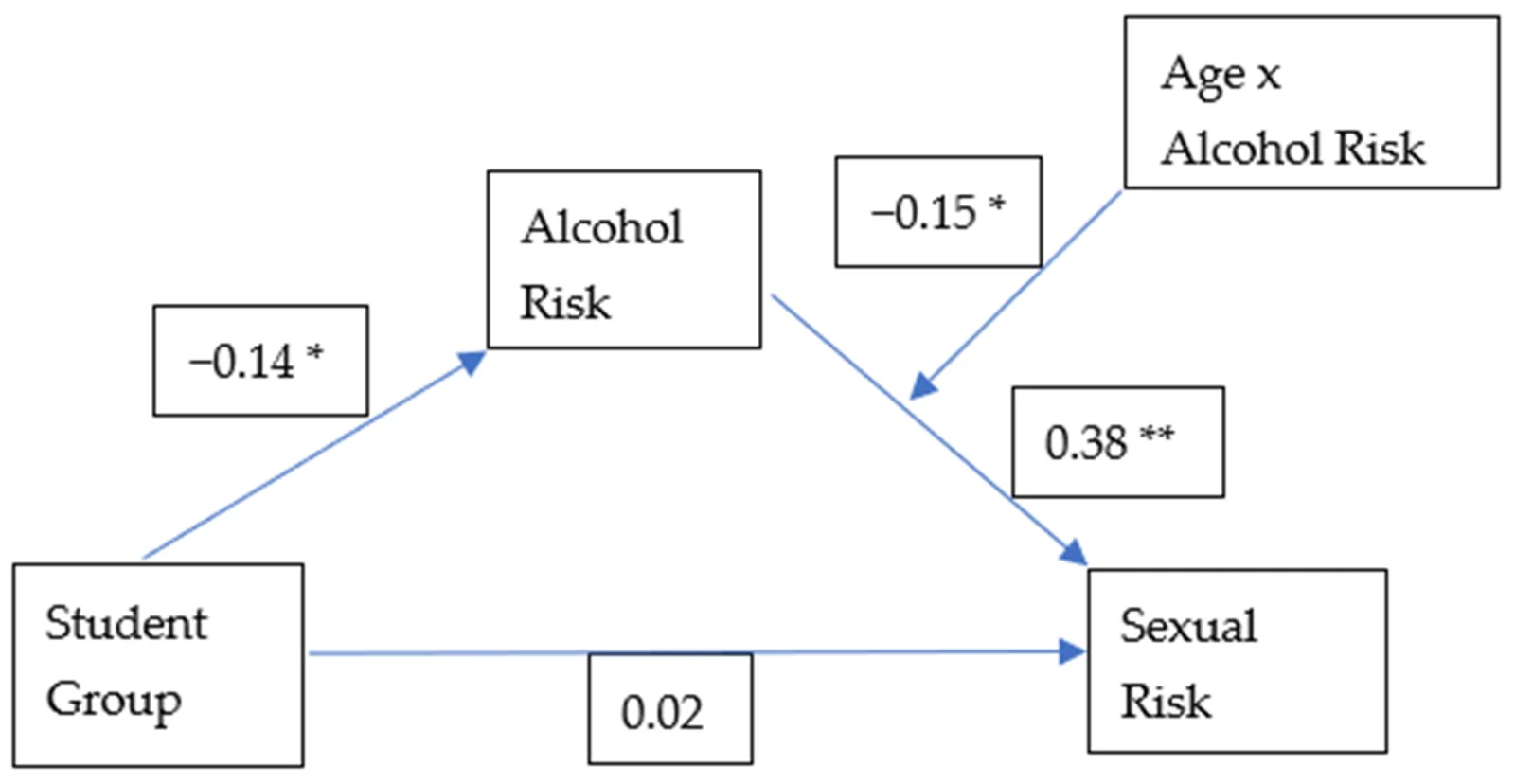
Path diagram with standardized coefficients. *n* = 3325. Student groups were coded as 1 = Australian-born domestic students, 2 = domestic students who were born in countries other than Australia, 3 = international students; * = *p* < 0.05; ** = *p* < 0.01.

As hypothesized via Hypotheses 1 and 2A, 2B, and 2C, there was no direct effect between group members and sexual risk (γ = 0.12, *p* = 0.28). However, there was full mediation via alcohol risk, meaning that there was an indirect effect between student group and sexual risk, which was mediated by alcohol risk. Namely, mediation was detected via paths from groups to risky drinking behaviour (γ = −0.71, *p* < 0.01) and from risky drinking behaviour to sexual risk (γ = 0.46, *p* < 0.01). Unstandardized coefficients for these paths are presented in Figure 1. Standardized coefficients with a path diagram are presented in Figure 2.

Furthermore, as an additional interesting effect, age moderated this indirect mediation effect, whereby the relationship between alcohol risk and sexual risk was strongest for younger participants relative to those near the middle or mean age range for the sample and older participants. Namely, conditional effects of alcohol risk in determining sexual risk were strongest for youngest participants (γ = 0.41, *p* < 0.01) and participants in the middle of the age range of the sample (γ = 0.35, *p* < 0.01), with older participants showing a significant but weaker effect (γ = 0.29, *p* < 0.01). Unstandardized coefficients for these paths are presented in Figure 1. The standardized coefficient for this moderation is presented within the path diagram in Figure 2.

The overall significant indirect effect of the entire model was also different at different levels of age, which is revealed in Table 3. This shows that the effect of the student group in determining sexual risk through alcohol risk was strongest for younger participants, moderate for age categories in the middle and weakest for older participants. The R Square for the overall model was significant at 0.08. Table 4 reports the overall model summary for the model displayed in Figure 1.

## 6. Discussion

This study aims to examine whether student group enrolment (Australian-born domestic, overseas-born domestic, and international students) is associated with harmful alcohol use, sexual risk-taking, and age, and to test a mediated moderation model in which harmful alcohol use mediates the relationship between student group membership and sexual risk-taking, with age as a second moderating factor.

Findings from the moderated mediation analysis found that group membership alone did not have a direct significant effect on sexual risk. However, this effect was fully mediated by harmful alcohol use, which suggests an indirect effect between group membership and sexual risk-taking behaviour. Furthermore, age moderated this indirect mediation effect, with the relationship between harmful drinking behaviour and sexual risk-taking behaviour being strongest for the mean age range of the sample (20 to 24 years old), compared with younger and older participants.

The results in the current study extend on past findings in the Australian university context, where variation in patterns of alcohol use and sexual risk-taking behaviours were found between international and domestic students [6,21]. More specifically, previous studies reporting international students studying at Australian universities identified lower alcohol consumption at harmful levels with less likelihood of experiencing alcohol-related harms, compared with domestic students [18,22]. Previous publications using the TSSRH survey have reported international students exhibit lower sexual risk-taking behaviours than their domestic counterparts [5,7]. The results from the current study, consistent with past research on international students, identified overall lower levels of sexual risk-taking behaviour and lower levels of harmful drinking behaviour, compared with domestic students.

Drawing from findings among international students in Australia, research suggests that acculturation stressors and time spent in Australia lead to students adapting to social expectations of increased alcohol consumption, which may differ from their ethnic heritage [21,24]. Differences in cultural norms in Western contexts have also been identified as a sexually liberating experience for international students, which allows for sexual exploration during their time abroad [9]. Asian and Sub-Saharan African international students in Australia were found to change their sexual behaviours and trial non-committal or premarital sex, associated with changes in cultural context between their home country and Australia [11,12]. Research has highlighted that lower levels of sexual risk-taking behaviour among international students should not be viewed as a constant state, as these student’s sexual behaviours may change over time due to associations with acculturation or psychological stressors associated with studying abroad [33].

Findings from the current research identify slightly lower levels of harmful alcohol use between overseas-born domestic students and Australian-born domestic students, with similar levels of sexual risk-taking behaviour between both groups. Overseas-born domestic students were however found to have higher levels of harmful drinking behaviour and sexual risk-taking behaviour, compared with international students. These findings may be explained by the aforementioned factors, such as time spent in Australia and acculturation factors. However, the lack of research on harmful drinking behaviours and sexual risk-taking behaviours among overseas-born domestic students in Australia that have been conducted supports the need for further research to determine the relationship between the variables for this group of students.

It is important to note, however, that there were no significant differences in sexual risk-taking behaviour between Australian-born domestic students, overseas-born domestic students, and international students. The relationship between sexual risk-taking behaviour and student group membership was fully mediated by harmful alcohol use, suggesting that differences in sexual risk-taking behaviours between student groups are primarily driven by differences in harmful alcohol use. These findings are consistent with the well-established link between alcohol use and sexual risk-taking behaviours among adolescents and young adults [32], with findings from this study extending this relationship to an Australian university context.

Similar to previous research showing 43% of students blamed alcohol use for regretful sexual experiences, and that Australian university students aged 18 to 30 years were at risk of higher alcohol consumption [18], the current study identifies students between the age of 20 to 24 years old are most likely to engage in harmful alcohol use and sexual risk-taking behaviour. The moderated mediation analysis further identifies age as a significant moderation factor in the relationship between risky drinking and sexual risk-taking behaviour, suggesting that younger age groups are more likely to engage in harmful alcohol use and sexual risk-taking behaviour.

## 7. Limitations

Limitations of the current study are that student responses were collected via a cross-sectional convenience sample recruited from sociocultural networks, and the survey was not translated into other languages. Accordingly, the model we present cannot be determined as causal, but rather reflecting associations within cross-sectional data. Considering that past research with refugee and migrant background populations have identified language barriers as a significant factor that leads to lower health literacy [34], it is possible that some of the terms or words used in the survey do not translate to certain cultural groups. This may have also impacted engagement with different cultural groups.

Another limitation of the current study is the measure of sexual risk behaviour which focuses on the mode of transmission, frequency of condom use, partnership type and number of partners; however, risk is also context-dependent. For example, research to develop a validated sexual risk measure found that measuring the frequency of condom use alone was not a reliable measure to distinguish between high- or low-risk behaviour participants [17]. Other contextual factors such as their own HIV/STI status, use of antiretroviral therapy and HIV Pre-Exposure Prophylaxis, and partnership type were important in determining risk between patient groups [17]. Additional considerations for sexual risk-taking behaviour within the context of Australian university students as a group when further developing the sexual risk behaviour measure may help to identify other risk factors associated with STI/HIV transmission in the university student context. Finally, we note that although the sample size employed in the current study was a particular strength of the findings, the effect sizes revealed in Figure 2 represent only small to modest effects.

### Directions for Future Research

While research has identified partnership status as an important risk factor for sexual risk-taking behaviours and STI transmission [35], there are also significant differences in the interpretation of partnership status [17,35]. It is possible that participants’ personal interpretations of sexual risk-taking behaviour items with regular partners may influence their survey responses. In future research, questions designed to check respondent interpretation of partnership status and risky sexual behaviour will be important to enhance the validity of the study conclusions. In addition, there are a host of psychological individual differences which could serve as useful correlates or even predictors of risky drinking behaviour and risky sexual behaviour. For example, elements of personal identity such as structure of identity have been strongly correlated with social decision-making, self-understanding, self-certainty, stability, and self-esteem [36] and can be most useful in predicting behaviours when individuals are going through significant life changes and transformations, such as entering university. Another important psychological individual difference, which has been shown to promote better social decision-making and emotional regulation, is practical wisdom [37]. Practical wisdom is particularly important under stressful life periods such as university studying; short forms for assessing practical wisdom are available and can be employed as a part of assessing wisdom within a few minutes during educational and health system practices [38]. Understanding these psychological and behavioural factors could also inform the development of practical, evidence-based interventions and policies, such as programs that address alcohol use alongside sexual health education, tailored specifically to different cultural backgrounds and age groups.

## 8. Conclusions

This study extends the established relationship between alcohol use and sexual risk-taking behaviours to an Australian university context and different groups of students (Australian-born domestic students, overseas-born domestic students, and international students) within Australia. Differences in sexual risk-taking behaviour between student membership groups are better explained when including harmful alcohol use in the analysis. This relationship is further moderated by age, with younger students being more likely to engage in harmful alcohol use and sexual risk-taking behaviours. Our findings also suggest that for overseas-born domestic students, increased time in Australia may be associated with increased harmful alcohol use and a subsequent increase in sexual risk-taking behaviour. These findings could help to inform sexual health promotion for university students, including the need to consider the needs of overseas-born domestic students as sperate to their Australian-born domestic and international student peers.

## Figures and Tables

**Table 1:** Sexual Risk Index and Alcohol Risk Index items by student group.

	Australian-Born Domestic Students *n* = 2530	Overseas-Born Domestic Students *n* = 349	International Students *n* = 446	All Students *n* = 3325
	Frequency/Percent	Frequency/Percent	Frequency/Percent	Frequency/Percent
Age 18–19 years	703 (27.8)	112 (32.1)	43 (9.6)	858 (25.8)
Age 20–24 years	1145 (45.3)	144 (41.3)	223 (50)	1512 (45.5)
Age 25–29 years	363 (14.3)	22 (6.3)	121 (27.1)	506 (15.2)
Age 30–34 years	131 (5.2)	23 (6.6)	46 (10.3)	200 (6.0)
Age 35–39 years	91 (3.6)	17 (4.9)	10 (2.2)	118 (3.5)
Age 40 years of age or older	97 (3.8)	31 (8.9)	3 (0.7)	131 (3.9)
	Mean (SD)	Mean (SD)	Mean (SD)	Mean (SD)
Sexual Risk Index	5.70 (4.40)	5.95 (5.00)	5.43 (4.32)	5.69 (4.45)
Alcohol Risk Index	6.14 (3.67)	5.89 (3.69)	4.59 (3.23)	5.91 (3.66)

Note: *n* = 3325.

**Table 2:** Correlations among study variables.

Variables	1	2	3	4
(1) Age	1			
(2) Student Group (By Birth Location)	0.07 **	1		
(3) Sexual Risk Index	−0.04 *	−0.01	1	
(4) Alcohol Risk Index	−0.08 **	−0.14 **	0.27 **	1

Note: ** *p* < 0.01, * *p* < 0.05; *n* = 3325.

**Table 3:** Conditional indirect effects of student group in determining sexual risk.

Age Group	Effect	BootSE	Boot LLCI	Boot ULCI
(1)	−0.29	0.04	−0.37	−0.21
(2)	−0.25	0.03	−0.31	−0.18
(3)	−0.21	0.03	−0.27	−0.15

Note: *n* = 3325. Age Group 1 = youngest; Age Group 2 = median age; Age Group 3 = oldest.

**Table 4:** Model summary.

R	R-Sq	MSE	F	df1	df2	*p*	Index of Moderated Mediation
0.28 **	0.08 **	18.28	70.95	4	3320	<0.01	0.04 [0.02, 0.07]

Note: ** *p* < 0.01, *n* = 3325.

## Data Availability

Data from this project is stored securely by the research team and is available for review upon request by appropriate fellow researchers.
